# Development of a Rapid Soil Water Content Detection Technique Using Active Infrared Thermal Methods for In-Field Applications

**DOI:** 10.3390/s111110114

**Published:** 2011-10-25

**Authors:** Francesca Antonucci, Federico Pallottino, Corrado Costa, Valentina Rimatori, Stefano Giorgi, Patrizia Papetti, Paolo Menesatti

**Affiliations:** 1 Agricultural Engineering Research Unit of the Agricultural Research Council (CRA-ING), Via della Pascolare 16, 00015, Monterotondo scalo (Rome), Italy; E-Mails: fedepall@yahoo.it (F.P.); corrado.costa@entecra.it (C.C.); valentina.rimatori@libero.it (V.R.); destratos@hotmail.com (S.G.); paolo.menesatti@entecra.it (P.M.); 2 Department of Economics, University of Cassino, Via Marconi 10, 03043, Cassino (FR), Italy; E-Mail: patrizia.papetti@libero.it

**Keywords:** soil moisture, Partial Least Squares, thermography, thermometry, sensor techniques, irradiance, heat dissipation

## Abstract

The aim of this study was to investigate the suitability of active infrared thermography and thermometry in combination with multivariate statistical partial least squares analysis as rapid soil water content detection techniques both in the laboratory and the field. Such techniques allow fast soil water content measurements helpful in both agricultural and environmental fields. These techniques, based on the theory of heat dissipation, were tested by directly measuring temperature dynamic variation of samples after heating. For the assessment of temperature dynamic variations data were collected during three intervals (3, 6 and 10 s). To account for the presence of specific heats differences between water and soil, the analyses were regulated using slopes to linearly describe their trends. For all analyses, the best model was achieved for a 10 s slope. Three different approaches were considered, two in the laboratory and one in the field. The first laboratory-based one was centred on active infrared thermography, considered measurement of temperature variation as independent variable and reported *r* = 0.74. The second laboratory–based one was focused on active infrared thermometry, added irradiation as independent variable and reported *r* = 0.76. The in-field experiment was performed by active infrared thermometry, heating bare soil by solar irradiance after exposure due to primary tillage. Some meteorological parameters were inserted as independent variables in the prediction model, which presented *r* = 0.61. In order to obtain more general and wide estimations in-field a Partial Least Squares Discriminant Analysis on three classes of percentage of soil water content was performed obtaining a high correct classification in the test (88.89%). The prediction error values were lower in the field with respect to laboratory analyses. Both techniques could be used in conjunction with a Geographic Information System for obtaining detailed information on soil heterogeneity.

## 1. Introduction

Recently, the need to measure in-field the variability of soil characteristics has increased following both sensor engineering developments, as well as the necessity to apply innovative crop management systems [1]. Changes in soil characteristics such as cation exchange capacity, organic carbon and water content may occur as the sampling point changes, even by few cm. A fine analysis carried out with conventional methods would require a lot of manual and laboratory work and incur high costs for the numerous samplings needed [2]. Researchers have investigated several approaches in order to automate these procedures [3] and to overcome the critical aspect of soil management in collecting representative samples [4]. For these reasons, methods increasing the acquisition of a high number of sample variables at a relatively low cost and time, such as vehicle-mounted optical sensing devices, represent promising application perspectives [5]. These multi-devices systems could include mobile instruments (*i.e.*, visible-near and near infrared spectrophotometers, infrared thermometers and thermocameras). These could be used to measure different surface-layers soil parameters such as reflectance, absorbance and temperature.

An important soil property is the spatial variation of water content measured at a proper depth and time [6]. The description of spatiotemporal soil water content (SWC) changes requires understanding of both spatial and time variability but results are relevant for many applicative agricultural contexts such as for example: trafficability, soil compactness and crop hydric stress [7]. Generally, the most common techniques to analyse SWC use punctual, destructive, expensive or time-consuming procedures [8,9], mainly based on opto-electronic, gravimetric, nuclear, electromagnetic, tensiometric and hygrometric processes [10]. Within the opto-electronic methods, near infrared (NIR) spectroscopy is one of the most used to calculate SWC in surface and subsurface layer, but its results show a tendency to underestimate values at higher water levels [11–13]. Another similar approach was carried out by Maltese *et al.* [14]. In this work the technological development of imaging sensors acquired in the visible (VIS), NIR and thermal infrared (TIR), renewed the research interest in setting up remote sensing based techniques aimed at retrieving SWC variability in the soil-plant-atmosphere system (SPA). The soil thermal inertia method (soil resistance to surrounding temperature change) is an additional method widely used to estimate soil moisture from TIR and VIS bands for bare soil [15,16]. This technique requires readily available soil characteristics such as soil texture and bulk density. Among the gravimetric methods, the oven-drying technique is probably the most widely used. This method is considered as the standard for the calibration of all other soil moisture determination techniques. Nevertheless, it has some disadvantages, being a destructive test requiring sample removal and making it impossible to measure the water content at exactly the same point at a later date [17]. Another method is neutron scattering. This method obtains a profile of moisture distribution but it has some disadvantages such as radiation hazards, insensitivity near soil surface, insensitivity to small variations in moisture content at different points, and variation in readings due to soil density variations, which may cause an error rate of up to 15% [18]. Among the electromagnetic techniques there are those that measure the soil electrical resistivity, obtaining hence its water content. In this case, the disadvantages regard the instable calibration over the time affected by ionic concentration and the cost of equipment [10].

Another widely used method for small spatial scale estimates of SWC is the measurement of soil thermal properties such as the heat dissipation technique and the heat pulse technique [19]. This method, contrary to the other previously reported ones, is non-destructive, and requires a small sample size which provides good spatial resolution, it is suitable for laboratory and field applications, does not need any calibration and conversely to the known electromagnetic techniques, it does not modify the soil’s electric properties. These are over a certain period of time permanently modified invalidating future readings [10]. These indirect methods exploit changes in soil thermal properties due to variation of SWC. In soil, the driving force which regulates its temperature is the water content, being its specific heat (*i.e.*, 1 J/g °C) higher than that of the other substances that make up the soil itself (0.19–0.35 J/g °C). In fact, the same amount of heat supplied to certain soil samples with different water contents can lead to different temperature differentials. Commercial heat dissipation sensors are broadly available. They basically consist in a heat source (usually a heated needle) and temperature sensors, immersed in a porous ceramic that equilibrates with the surrounding soil at a given water content. The needle is heated and the rate of heat dissipation is measured by the temperature sensors [20]. However, sensor use is limited by the need of calibration for any type of soil and by the long time to reach hydraulic equilibrium with the surrounding soil. The time required to reach the hydraulic equilibrium between heat dissipation sensors and soil depends on both the magnitude of the SWC and the hydraulic conductivity. Typically this equilibration time is on the order of minutes or tens of minutes [21].

In order to overcome the limits of heat dissipation sensors, in this study we propose the use of a new technique based on the same underlying theory of the heat dissipation methods. Unlike heat dissipation sensors, we propose to directly measure temperature changes of soil samples, after heating, by using active infrared thermography and thermometry. The assumption is that these techniques could lead to the development of a faster SWC measurement system and could represent informative and non-destructive tools to remotely assess the dynamic variation of soil temperature [22,23]. Moreover, these could be implemented on vehicle-mounted systems to shorten sampling time and the amount of soil surveyed. The main principle of these applications concerns the measurement of the thermal infrared spectrum of electromagnetic radiation emitted by soil samples depending on their temperature [24,25]. For in-field applications this technique should measure surface soil (0–60 cm) temperature, that is influenced by soil-atmosphere interactions. This aspect makes unsuitable the use of calibration curves to relate temperature to SWC as physical or empirical relationships, which describe all the soil-atmosphere interactions. In fact the general model describing the soil-atmosphere interaction is given by the energy balance equation [26]:

(1) $$R_{n}+M-H-\lambda E=G$$

where R_n_ is the net radiation at soil surface, M represents the supply of energy to the surface by metabolism or absorption of energy by photosynthesis, H is the sensible heat flux, λE is the latent heat flux by evapotranspiration and G is the soil heat flux.

Adapting the energy balance Equation (1) to the proposed study and analyzing the water content on a bare soil after primary tillage and exposed to soil irradiance, the M becomes negligible and G is equal to:

(2) $$G\;=\;G_{s}+G_{1}$$

where G_s_ is the heat variation of soil surface and G_l_ the heat flux in the soil by contact. The surface thermal variation will be related to G_s_, H, λE and G_l_. In this case, these parameters will be dependent on agro-pedological and meteorological parameters such as air temperatures and humidity, SWC, irradiance, wind regimes, soil water potential and soil roughness. The deterministic modelling of the environmental variables influencing the physical process which is developing in such a short time of analysis (few seconds) would have been very complex.

For the above mentioned reasons the system could be approached in a statistical way and the estimation of SWC innovatively implemented by using a multivariate analysis [27,28], taking into consideration different soil thermal properties and meteorological parameters as input variables. Differently from deterministic models, stochastic ones do not explain the underlying physical processes generating the observations and the model randomness. Modeling spatiotemporal distributions, resulting from dynamic processes and evolving in both space and time, is critical in hydrology and soil science. Statistical spatiotemporal models provide a probabilistic framework for data analysis based on joint spatial and temporal dependence among observations [7].

In this study, a multivariate statistic approach (Partial Least Squares regression, PLS, and Discriminant Analysis, PLSDA) is used to estimate the SWC with active infrared thermal methods by warming up and measuring, at different time steps, several non-factorial soil samples with different water contents. Three different hypotheses were considered, two in the laboratory and one in the field. The laboratory experiments were carried out to determine the best performing one. The latter was then chosen in order to be applied in-field. The first one tested in the laboratory is based on active infrared thermography, which considers only the measurement of temperature variation as independent (observed) variable. The second one examined in the laboratory added the irradiation of soil samples as independent variable and it was based on active infrared thermometry. Finally the in-field experiment was based on active infrared thermometry and also considered some meteorological parameters as independent variables (*i.e.*, air temperature and relative humidity, wind speed and irradiance at soil surface).

## 2. Experimental Section

### 2.1. Laboratory Analysis

In order to develop models for the statistical interpretation of the phenomenon, according to the previously indicated thermo-physical context, a series of progressive laboratory tests were performed. These laboratory tests were developed to highlight the limits and possibilities of the techniques and chose among them the most suitable one for an in-field application.

The experimental laboratory protocol consists in warming up soil samples with different initial temperatures and water contents and in measuring for a few seconds the dynamic temperature variations. This investigation was carried out in two different steps: the first with an infrared thermocamera considering as dependent variable the percentage of water content and as independent ones the initial soil temperature and the exposition time at constant irradiance.

In a second step, an infrared thermometer was used to simplify the measuring system by introducing among the independent variables also the irradiance produced by photographic bulbs (200 W and 2,800 K) to approach in-field applications. In both cases, air temperature and air relative humidity were considered as constant.

#### 2.1.1. Thermographic Analysis

Soil samples, number of soil samples (N) = 250, were collected from the CRA-ING experimental field (Lat. 42°06′11.00″N, Long. 12°37′40.81″E) at a depth of 30 cm. The operative soil status being normally unknown, different initial levels of water content and temperature were achieved by hydrating, dehydrating, warming up (stove, 40–60 °C; controlled environment, 20–25 °C) and cooling down (fridge, 2–4 °C) different plastic trays (20 × 30 cm) previously filled with soil samples.

The dynamic variation of sampled soil temperature was identified by an operator analyzing a specific thermal image area called the Region Of Interest (ROI). The temperature values were collected at four different intervals: 0, 3, 6 and 10 s. The water content (%) was expressed gravimetrically as percentage of grams (g) of water on g of dry soil (θ g, water g/dry soil g). The water content reference measurements of samples were obtained through the official oven-drying gravimetric technique [17], by placing the sample in an oven at 105 °C until stabilization of weight.

**Figure 1.**
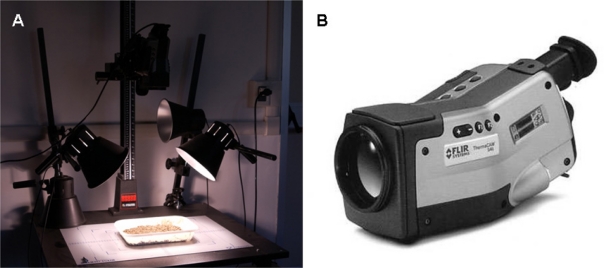
**(A)** Thermographic laboratory analysis system. Special photographic bulbs heating the soil samples in apposite plastic trays (20 × 30 cm) for the active infrared thermographic analysis. **(B)** Thermocamera FLIR (S40).

The soil surface temperature dynamic variation was acquired using a FLIR (S40) thermocamera [Figure 1(A,B)] with the following characteristics: detector type, Focal Plane Array (FPA) uncooled microbolometer; Field Of View (FOV), 24° at distance of 1 m the FOV is equal to 0.42 × 0.31 m; Instantaneous Field Of View (IFOV), 1.3 mrad (the theoretical FOV of one pixel); image frequency, 60 Hz; spectral range, 7.5 to 13 μm; focus, automatic or manual; thermal sensitivity 50/60 Hz, 0.08 °C at 30 °C; temperature range −40–+120 °C. The emissivity (ɛ), the capability of an object to adsorb or emit the thermal radiation, for the soil was set equal to 0.96 [26].

#### 2.1.2. Thermometric Analysis

As in the previous case, soil samples (N = 50) were collected in the CRA-ING experimental field at a depth of 30 cm. In addition to different initial levels of water content and temperature, different irradiance values were considered for all the samples. Irradiance was measured by a radiation sensor whose sensible element is a photodiode that converts incident radiation into a voltage (LP-9021 RAD, Delta Ohm, Padova, Italy). The signal is then acquired by a portable microprocessor-controlled multifunction quantum-photo-radiometric indicator with LCD indication (HD-9021, Delta Ohm). The sensor measures the flux of incident radiation in the spectral region spanning from 450 nm to 950 nm, ranging from 0 to 2,000 W/m^2^ and having a precision of ±3.5%. The portable indicator has a resolution of 0.1 W/m^2^ for values minor than 200 W/m^2^ and 1 W/m^2^ for values greater than 200 W/m^2^.

Soil surface temperature dynamic variation was measured at four intervals (0, 3, 6 and 10 s) by an infrared thermometer measuring the amount of radiant energy emitted by the samples (IRtec P500, Eurotron, Milano, Italy). The instrument has a measurement range from −30 °C to 930 °C, a resolution of 0.1 °C and an accuracy of ±1% + 1 °C. Also in this case the water content reference measurements of samples were obtained through the oven-drying gravimetric technique [11].

### 2.2. In-Field Analysis

**Figure 2.**
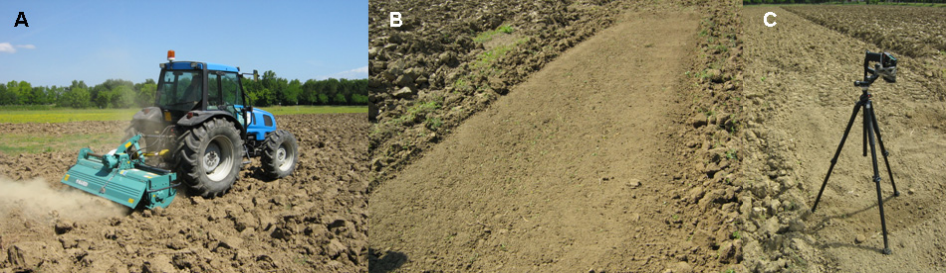
**(A)** Soil deep ploughing (60 cm); **(B)** Soil after primary tillage (15 cm); **(C)** Temperature dynamic variations acquisition through the infrared thermometer.

Soil samples (N = 40) were collected in the CRA-ING experimental field facilities during three different days and times in order to obtain a high SWC and solar irradiance variability. The samples were collected on the surface of bare soil after deep ploughing (60 cm) [Figure 2(A)] and primary milling (15 cm) [Figure 2(B)]. The measurements were carried out after ploughing just for practical reasons but the models are meant to work in pair with any soil baring system. The temperature dynamic variations were measured at four intervals (0, 3, 6 and 10 s) by the infrared thermometer [Figure 2(C)] on 40 different surface points of the bare soil. The temperature variation was achieved by solar irradiance and measured with the radiation sensor. At the same time air temperature and relative humidity and wind speed were collected with a weather station (Vantage Pro2™, Davis Instruments Corp., Hayward, CA, USA). The water content reference measurements were obtained through the oven-drying gravimetric technique [17], catching the first 5 cm of the bare analyzed soils and bringing them in laboratory after temperature acquisition.

### 2.3. Datasets Creation and Statistical Analysis

For the datasets creation, the temperature dynamic variations were collected at four intervals (0, 3, 6 and 10 s), named hereafter t_0_, t_3_, t_6_ and t_10_, respectively. For the presence of different specific heats between water and soil, the analyses were regulated with the addition of slopes obtained by interpolation values for each interval (t_3_ slope, t_6_ slope and t_10_ slope). The t-slopes were calculated from the initial temperature (t_0_) to the final one, including all the internal steps (*i.e.*, t_10_ slope was calculated from the t_0_, t_3_, t_6_ and t_10_ values).

Three different datasets were hence created. The first in the laboratory method based on the active infrared thermography considered only the measurement of temperature variation as independent (observed) variable. The second laboratory one added the irradiation of soil samples as independent variable and it was based on active infrared thermometry. Finally, the in-field experiment was based on active infrared thermometry and it also considered as independent variables some meteorological parameters (*i.e.*, air temperature and relative humidity, wind speed and irradiance at soil surface).

The SWC estimation in all the analysis, both in laboratory (*i.e.*, thermographic and thermometric) and in the field (*i.e.*, thermometric), was carried out by multivariate PLS regression analysis [29] on the basis of thermal data collected at the intervals t_3_, t_3_ slope, t_6_, t_6_ slope, t_10_ and t_10_ slope for the thermographic analysis and only at the interval t_10_ slope for both laboratory and in-field thermometric analysis taking a temperature reading every second. For these two last analyses only the t_10_ slope was considered because it performed better than the t_3_ and t_6_ ones. This is due to the presence in-field of the environmental variables producing noises that can be lowered with a longer acquisition.

The procedure of PLS [28,30] was elaborated using the PLS Toolbox in MATLAB V7.0 R14 (The Math Works, Natick, MA, USA) and included the following steps: (1) extraction of raw thermal data (X-block variables); (2) extraction of measured SWC (Y-block variables); (3) data fusion of the two dataset (Y and X-block) in one analysis dataset (AS); (4) the sample set partitioning based on joint x-y distances (SPXY) [29–31] separation of the AS into two subsets, one (MS) for the model (85% of AS) and one (TS) for the external validation test (15% of AS); (6) application of different pre-processing algorithms (Table 1) to X-block and Y; (7) application of chemometric technique (PLS): modeling and testing; (8) calculation of efficiency parameter of prediction.

The predictive ability of the model is partially dependent on the number of Latent Vectors (LV) used and was assessed by the prediction efficiency parameters: Root Mean Square Error (RMSE), Standard Error of Prevision (SEP) and correlation coefficient (*r*) between observed and predicted values. Finally, we recorded the Ratio of Percentage Deviation (RPD), which is the ratio of the standard deviation of the laboratory measured (reference) data to the RMSE [32]. It is the factor by which the prediction accuracy has been increased compared with using the mean of the original data. The model chosen was for the number of LV that yielded the highest *r*, minimum SEP for predicted and observed water content and maximum RPD.

In order to obtain more general and wide (*i.e.*, mapping) estimations of soil water content characteristics in the in-field analysis a Partial Least Squares Discriminant Analysis (PLSDA) [33] was performed. This model considered three different classes of water content (low < 11%; 11% < medium < 14% and high > 14%) and calculated a prediction probability and a classification threshold for each class modelled. The samples from each class were subdivided in two subsets: (i) 75% of samples for the class modelling and validation; (ii) 25% of specimens for the independent test, optimally chosen with the Euclidean distances based on the algorithm of Kennard and Stone [34] that selects objects without the a priori knowledge of a regression model (the hypothesis of a flat distribution of the data is preferable for a regression model). This analysis provided the percentage of correct classifications and the modelling efficiency in terms of sensitivity and specificity parameters where the first represents the percentage of the samples of a category accepted by the class model and the second the percentage of the samples of the categories different from the modelled one, rejected by the class model.

## 3. Results

### 3.1. Laboratory Results

#### 3.1.1. Thermographic Results

Table 2 shows the results of PLS for the prediction of SWC through thermographic analysis for the three time intervals (t_3_, t_6_ and t_10_) and for the slopes obtained by value interpolation for each interval (t_3_ slope, t_6_ slope and t_10_ slope) with maximum *r* and RPD (calculated to RMSE of test subset) and minimum SEP for the calculation of water content.

#### 3.1.2. Thermometric Results

**Figure 3.**
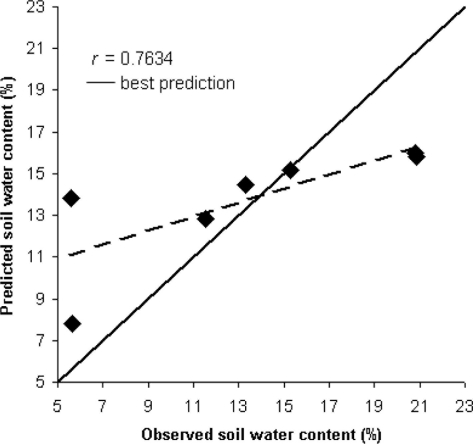
Regression between observed and predicted values of soil water content for the intervals t_10_ slope in the independent test for the thermometric analysis (*i.e.*, 15% of whole sample dataset).

Table 3 reports the results of PLS for the prediction of SWC through thermometric analysis only for the t_10_ slope interval, assessed with maximum *r* and RPD (*i.e.*, calculated to RMSE of test subset) and minimum SEP for the calculation of water content. Figure 3 shows the regression between observed and predicted values relatively to the prediction for the prediction of SWC through thermometric analysis in the independent test for t_10_ slope.

### 3.2. In-Field Results

Table 4 reports the results for the prediction of SWC through thermometric analysis performed in-field for the only interval t_10_ slope. Table 5 reports the results of the PLSDA for the prediction of SWC through thermometric analysis performed in-field for the only interval t_10_ slope considering three different classes of SWC (low < 11%; 11% < medium < 14% and high > 14%).

## 4. Discussion and Conclusions

As reported by Bittelli [20], SWC estimation is necessary for different applications, ranging from large-scale calibration of global-scale climate models to field monitoring in agricultural and horticultural systems. The proposed laboratory and in-field methods concern the development of non-destructive and rapid SWC estimations using active infrared thermography and thermometry in combination with multivariate statistical analysis (PLS). The main principle of these applications regards the measurement of the thermal infrared spectrum of electromagnetic radiation emitted by samples depending on their dynamic temperature variation achieved by heating soil [24,25]. In this work the statistical modeling based on a variant of the heat dissipation method occurs efficacy in all the experimental analysis overcoming the limits of the heat dissipation sensors measuring directly the temperature dynamic variation of soil samples after heating.

Generally among the results shown by both laboratory and in-field applications, the best performing models were the t_10_ slope ones. The laboratory analysis showed that active infrared thermometry performed better than thermography, probably due to the variable represented by the irradiance present in the statistical model. This latter increased the correlation coefficient (*r*) in the independent test (0.7417 for thermography; 0.7634 for thermometry) but it especially decreased both SEP and RMSE (12.314 and 15.512 for thermography; 4.5316 and 4.2138 for thermometry). The RPD values remained instead similar (1.4524 for thermography and 1.2868 for thermometry).

Since the best performing laboratory model was thermometry, we have chosen to use only this methodology for the in-field applications in order to measure SWC from the dynamic variation of surface temperature after deep ploughing and primary tillage. Both ploughing and tillage were used only for practical reasons. This in-field measurement is influenced by soil-atmosphere interactions as reported by Campbell and Norman [26]. This makes the use of calibration curves unsuitable temperature to SWC as physical or empirical relationships which describe all the soil-atmosphere interactions. Moreover, for a correct infrared thermal measurement the estimation of emissivity is very important. As reported by Schmugge [35] the rate of soil emissivity is a function of its texture and it is greater for lighter sandy and smaller for heavier clayey soils and it is reduced by surface features, such as roughness and vegetation cover. In this study, to overcome the heterogeneity of soil in terms of surface, temperature and water content we analyzed a great number of diverse non-factorial samples, mostly in the laboratory. Adapting the energy balance to the proposed study, this becomes dependent by agro-pedological and meteorological parameters such as air temperatures and humidity, SWC, irradiance, wind regimes, soil water potential and soil roughness making the system approachable in a statistic way instead of deterministic one. Thus, the estimation of SWC was developed by using a multivariate analysis by taking into consideration different soil thermal properties and meteorological parameters as input variables. This statistical approach provided a probabilistic framework for data analysis, as based on joint spatial and temporal dependence among observations [7,27,28].

In particular, the proposed in-field method performed worse than the laboratory ones (*r* = 0.6063 and RPD = 0.9742), but reported very low prediction error values (SEP = 3.6123 and RMSE = 3.3194). In this case, the lower performance could be related to the measurement of thermo-hygrometer variables. In fact, the air temperature and relative humidity and wind speed were not punctually collected (*i.e.*, we used a weather station). In order to obtain more general and wide (*i.e.*, mapping) estimations of SWC in in-field analysis a PLSDA was performed. In this case three different classes of water content (low <11%; 11% < medium < 14% and high >14%) were considered and a prediction probability and a classification threshold were calculated for each class modelled. The results showed a higher percentage of the mean correct classification both in the model (86.349%) and in the test (88.889%) with respect to the PLS. This classificatory model using a multivariate statistical approach as shown above can discriminate among close classes of SWC. This proves as such a technique is capable of a fine discrimination with respect to a simple linear modelling approach. This in-field gives the opportunity to choose properly the best trafficability and soil workability.

Therefore, relationships between soil water and environmental factors need to be studied over wider time- and spatial-scales [36]. However, these could be implemented on vehicle-mounted systems to shorten sampling time and amount of soil needed. Moreover, it could be possible to obtain temperature mapping and consequently water content directly in field of any particular region of interest with fast response times, which is not presently possible with thermocouples or other temperature sensors (*i.e.*, these can only measure spot data). In addition the repeatability of these measurements is high and it does not require an illumination source, unlike other systems [37].

Finally, both thermography and thermometry are of fast execution and could produce highly informative results if paired with a Geographic Information System (GIS). Also, as reported by Schmidhalter *et al.* [38], these could be applied on site-specific management tasks, as required in precision farming to obtain detailed information about the heterogeneity of soil. Moreover, the proposed methodologies resulted very interesting for the limited time of exposure to the heat, needed to obtain results on dynamic temperature variation, making these implementable on commercial machine systems for very expeditious in-field applications.

## Figures and Tables

**Table 1.** List of the different X and Y pre-processing techniques applied in the analysis.

**Label**	**Description**

None	No pre-processing
Baseline	Baseline (Weighted Least Squares)
Abs	Takes the absolute value of the data
Autoscale	Centres columns to zero mean and scales to unit variance
Detrend	Remove a linear trend
Groupscale	Group/block scaling
mean center	Center columns to have zero mean
median centre	Centre columns to have zero median
Normalize	Normalization of the rows
SNV	Standard Normal Deviate
Centering	Multiway Center

**Table 2.** Partial Least Squares (PLS) results for the prediction of soil water content (SWC) obtained with laboratory thermographic analysis for the three time intervals (t_3_, t_6_ and t_10_) and for the slopes obtained by values interpolation for each interval (t_3_ slope, t_6_ slope and t_10_ slope). The table reports n° of Latent Vectors (LV); first and second pre-processing for the X-block and one for the Y-block; the correlation coefficient (*r*); the Ratio of Percentage Deviation (RPD); the Standard Error of Prevision (SEP) and the Root Mean Square Error (RMSE) for the model and test.

**Parameters**	**t_3_**	**t_3_ slope**	**t_6_**	**t_6_ slope**	**t_10_**	**t_10_ slope**

**MODEL (85%)**						

**n° LV**	3	3	3	2	10	9
**First pre-processing X-block**	autoscale	none	autoscale	autoscale	autoscale	median center
**Second pre-processing X-block**	normalize	none	none	none	median center	none
**Pre-processing Y-block**	median center	autoscale	none	autoscale	none	median center
***r* (observed *vs.* predicted)**	0.3051	0.5765	0.6016	0.6113	0.7524	0.7756
**RPD**	1.0476	1.2209	1.209	1.2606	1.5133	1.5804
**SEP**	15.093	12.929	13.045	12.37	10.323	9.9083
**RMSE**	16.125	12.898	21.675	12.341	10.301	9.8858

**TEST (15%)**						

***r* (observed *vs.* predicted)**	0.2993	0.5198	0.5784	0.542	0.7227	0.7417
**RPD**	1.0322	1.1013	1.209	1.129	1.2163	1.4524
**SEP**	16.682	15.438	15.407	15.335	14.729	12.314
**RMSE**	19.444	15.286	27.478	16.258	26.281	15.512

**Table 3.** Results of Partial Least Squares (PLS) for the prediction of soil water content (SWC) obtained with laboratory thermometric analysis for the interval t_10_ slope. In the table are reported: n° of Latent Vectors (LV); first and second pre-processing for the X-block and one for the Y-block; the correlation coefficient (*r*); the Ratio of Percentage Deviation (RPD); the Standard Error of Prevision (SEP) and the Root Mean Square Error (RMSE) for the model and test.

**Parameters**	**t10 slope**

**MODEL (85%)**	

**n° LV**	4
**First pre-processing X-block**	autoscale
**Second pre-processing X-block**	none
**Pre-processing Y-block**	autoscale
***r* (observed *vs.* predicted)**	0.7095
**RPD**	1.4024
**SEP**	2.125
**RMSE**	2.1001

**TEST (15%)**	

***r* (observed *vs.* predicted)**	0.7634
**RPD**	1.2868
**SEP**	4.5316
**RMSE**	4.2138

**Table 4.** Results of Partial Least Squares (PLS) for the prediction of SWC obtained with thermometric analysis performed in field for the interval t_10_ slope. In the table are reported: n° of Latent Vectors (LV); first and second pre-processing for the X-block and one for the Y-block; the correlation coefficient (*r*); the Ratio of Percentage Deviation (RPD); the Standard Error of Prevision (SEP) and the Root Mean Square Error (RMSE) for the model and test.

**Parameters**	**t10 slope**

**MODEL (85%)**	

**n° LV**	5
**First pre-processing X-block**	mean center
**Second pre-processing X-block**	baseline
**Pre-processing Y-block**	autoscale
***r* (observed *vs.* predicted)**	0.6383
**RPD**	1.2803
**SEP**	1.6924
**RMSE**	1.6726

**TEST (15%)**	

***r* (observed *vs.* predicted)**	0.6063
**RPD**	0.9742
**SEP**	3.6123
**RMSE**	3.3194

**Table 5.** Results of Partial Least Squares Discriminant Analysis (PLSDA) for the in-field prediction of SWC obtained with thermometric analysis for the interval t_10_ slope considering three different classes of soil water content (SWC) (low < 11%; 11% < medium < 14% and high > 14%). N is the number of samples; n° units (Y-block) is the number of units to be discriminated by the PLSDA; n° LV is the number of latent vectors. Random Probability (%) is the probability of random assignment of an individual into a unit.

**Parameters**	**t10 slope**
**N (Low SWC < 11%)**	13
**N (11% < Medium SWC < 14%)**	19
**N (High SWC > 14%)**	9
**n° units (Y-block)**	3
**n° LV**	6
**% Cumulated Variance X-block**	100
**Mean Specificity (%)**	89.033
**Mean Sensitivity (%)**	86.667
**Random Probability (%)**	33.333
**Mean Classification Error (%)**	12.143
**Mean Correct Classification Model (%)**	86.349
**Mean Correct Classification Test (%)**	88.889
